# [μ-Bis(diphenyl­arsino)methane-1:2κ^2^ *As*:*As*′][bis­(4-methoxy­phen­yl)phenyl­phosphine-3κ*P*]-nona­carbonyl-1κ^3^ *C*,2κ^3^ *C*,3κ^3^ *C*-*triangulo*-tri­ruthenium(0) dichloro­methane 0.15-solvate

**DOI:** 10.1107/S1600536810001194

**Published:** 2010-01-30

**Authors:** Omar bin Shawkataly, Imthyaz Ahmed Khan, Chin Sing Yeap, Hoong-Kun Fun

**Affiliations:** a Chemical Sciences Programme, School of Distance Education, Universiti Sains Malaysia, 11800 USM, Penang, Malaysia; b X-ray Crystallography Unit, School of Physics, Universiti Sains Malaysia, 11800 USM, Penang, Malaysia

## Abstract

The asymmetric unit of the title *triangulo*-triruthenium compound, [Ru_3_(C_25_H_22_As_2_)(C_20_H_19_O_2_P)(CO)_9_]·0.15CH_2_Cl_2_, contains one mol­ecule of the *triangulo*-triruthenium complex and one partially occupied dichloro­methane solvent mol­ecule. The dichloro­methane solvent lies across a crystallographic inversion center leading to the mol­ecule being disordered over two positions of equal occupancy. The bis­(diphenyl­arsino)methane ligand bridges an Ru—Ru bond and the monodentate arsine ligand bonds to the third Ru atom. Both the arsine ligands are equatorial with respect to the Ru_3_ triangle. In addition, each Ru atom carries one equatorial and two axial terminal carbonyl ligands. The three phospho­rus-bound benzene rings make dihedral angles of 72.7 (3), 80.9 (3) and 70.8 (2)° with each other. The dihedral angles between the two benzene rings are 79.9 (3) and 81.5 (2)° for the two diphenyl­arsino groups.

## Related literature

For general background to *triangulo*-triruthenium derivatives, see: Bruce *et al.* (1985 ▶, 1988*a* ▶,*b* ▶). For related structures, see: Shawkataly *et al.* (1998 ▶, 2004 ▶, 2009 ▶). For the synthesis of μ-bis­(diphenyl­arsino)methane­deca­carbonyl­triruthenium(0), see: Bruce *et al.* (1983 ▶).
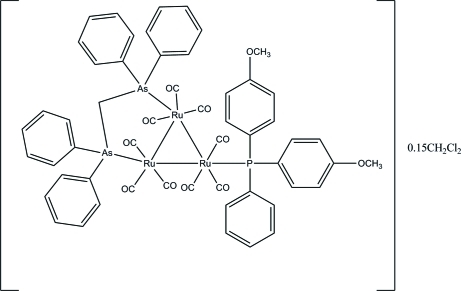


## Experimental

### 

#### Crystal data

[Ru_3_(C_25_H_22_As_2_)(C_20_H_19_O_2_P)(CO)_9_]·0.15CH_2_Cl_2_
*M* *_r_* = 1362.63
Monoclinic, 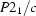

*a* = 13.3723 (4) Å
*b* = 16.9461 (6) Å
*c* = 25.0956 (8) Å
β = 93.293 (2)°
*V* = 5677.5 (3) Å^3^
*Z* = 4
Mo *K*α radiation
μ = 2.04 mm^−1^
*T* = 296 K
0.31 × 0.21 × 0.16 mm

#### Data collection

Bruker SMART APEXII CCD area-detector diffractometer
Absorption correction: multi-scan (*SADABS*; Bruker, 2005 ▶) *T*_min_ = 0.571, *T*_max_ = 0.741
67215 measured reflections
16557 independent reflections
10503 reflections with *I* > 2σ(*I*)
*R*_int_ = 0.041

#### Refinement

*R*[*F* ^2^ > 2σ(*F* ^2^)] = 0.039
*wR*(*F* ^2^) = 0.121
*S* = 1.02
16557 reflections
654 parameters
3 restraints
H-atom parameters constrained
Δρ_max_ = 1.00 e Å^−3^
Δρ_min_ = −0.38 e Å^−3^

### 

Data collection: *APEX2* (Bruker, 2005 ▶); cell refinement: *SAINT* (Bruker, 2005 ▶); data reduction: *SAINT*; program(s) used to solve structure: *SHELXTL* (Sheldrick, 2008 ▶); program(s) used to refine structure: *SHELXTL*; molecular graphics: *SHELXTL* software used to prepare material for publication: *SHELXTL* and *PLATON* (Spek, 2009 ▶).

## Supplementary Material

Crystal structure: contains datablocks global, I. DOI: 10.1107/S1600536810001194/tk2608sup1.cif

Structure factors: contains datablocks I. DOI: 10.1107/S1600536810001194/tk2608Isup2.hkl

Additional supplementary materials:  crystallographic information; 3D view; checkCIF report

## Figures and Tables

**Fig. 1.**
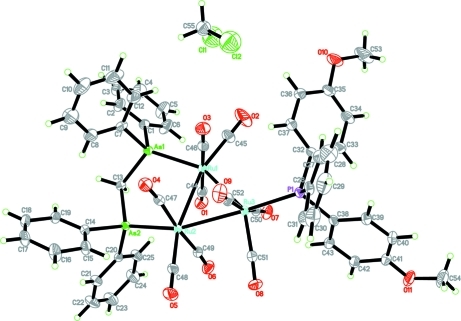
The molecular structure of the title compound with 20% probability ellipsoids for non-H atoms.

**Fig. 2.**
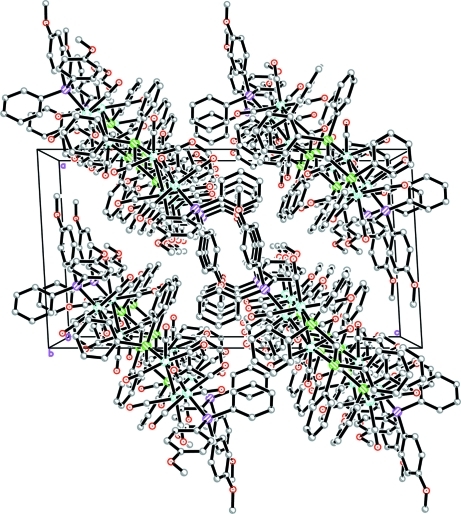
The crystal packing of the title compound, viewed down the b axis, showing the molecules stacked along b axis. Hydrogen atoms not involved in the hydrogen-bonding (dashed lines) and the solvent molecules have been omitted for clarity.

## supplementary crystallographic information

### Comment

*Triangulo*-triruthenium clusters are known for their interesting structural variations and related catalytic activity. A large number of substituted derivatives, Ru_3_(CO)_12-n_*L*_n_ (*L* = group 15 ligand) have been reported (Bruce *et al.*, 1988*a*,b; Bruce *et al.*, 1985). As part of our study on the substitution of transition metal-carbonyl clusters with mixed-ligand complexes, we have published several structures of *triangulo*-triruthenium-carbonyl clusters containing mixed P/As and P/Sb ligands (Shawkataly *et al.*, 1998, 2004, 2009). Herein we report the synthesis and structure of title compound.

The asymmetric unit consists of one molecule of the *triangulo*-triruthenium complex and a 15% partially occupied molecule of dichloromethyl solvent (Fig. 1). The dichloromethyl solvent lies across a crystallographic inversion center leading to the molecule being disordered over two positions of equal occupancy. The bond lengths and angles of title compound are comparable to those found in related structure (Shawkataly *et al.*, 2009). The bis(diphenylarsino)methane ligand bridges the Ru1—Ru2 bond and the monodentate arsine ligand bonds to the Ru3 atom. Both the arsine ligands are equatorial with respect to the Ru_3_ triangle. Additionally, each Ru atom carries one equatorial and two axial terminal carbonyl ligands. The three phosphine-substituted benzene rings make dihedral angles (C26–C31/C32–C37, C26–C31/C38–C43 and C32–C37/C38–C43) of 72.7 (3), 80.9 (3) and 70.8 (2)° with each other respectively. The dihedral angles between the two benzene rings (C1–C6/C7–C12 and C14–C19/C20–C25) are 79.9 (3) and 81.5 (2)° for the two diphenylarsino groups respectively. In the crystal structure, the molecules are stacked along the *b* axis (Fig. 2).

### Experimental

All manipulations were performed under a dry, oxygen-free dinitrogen atmosphere using standard Schlenk techniques, all solvents were dried over sodium and distilled from sodium benzophenone ketyl under nitrogen. Bis(4-methoxyphenyl)phenylphosphine (Maybridge) was used as received and µ-bis(diphenylarsino)methanedecacarbonyltriruthenium(0) was prepared by reported procedures (Bruce *et al.*, 1983). The title compound was obtained by refluxing equimolar quantities of Ru_3_(CO)_10_(µ-Ph_2_AsCH_2_AsPh_2_) (105.5 mg, 0.1 mmol) and bis(4-methoxyphenyl)phenylphosphine (32.23 mg, 0.1 mmol) in hexane under nitrogen atmosphere. Crystals were grown by slow solvent / solvent diffusion of CH_3_OH into CH_2_Cl_2_.

### Refinement

All hydrogen atoms were positioned geometrically and refined using a riding model with C—H = 0.93–0.97 Å and *U*_iso_(H) = 1.2 or 1.5 *U*_eq_(C). A rotating group model was applied for the methyl groups. The dichloromethane molecule is under constrained with fixed distance of 1.70 Å between C55 and Cl1/Cl2 and 2.94 Å between Cl1 and Cl2. The refined site occupancies of the dichloromethane molecule are fixed to be 0.15 at final refinement. The dichloromethane molecule is refined isotropically. The maximum and minimum residual electron density peaks of 1.00 and -0.38 e Å^-3^, respectively, were located 1.11 Å and 0.38 Å from the Cl2 and C36 atoms, respectively.

### Crystal data

**Table:**

[Ru_3_(C_25_H_22_As_2_)(C_20_H_19_O_2_P)(CO)_9_]·0.15CH_2_Cl_2_	*F*(000) = 2689
*M**_r_* = 1362.63	*D*_x_ = 1.594 Mg m^−^^3^
Monoclinic, *P*2_1_/*c*	Mo *K*α radiation, λ = 0.71073 Å
Hall symbol: -P 2ybc	Cell parameters from 9029 reflections
*a* = 13.3723 (4) Å	θ = 2.5–27.7°
*b* = 16.9461 (6) Å	µ = 2.04 mm^−^^1^
*c* = 25.0956 (8) Å	*T* = 296 K
β = 93.293 (2)°	Block, purple
*V* = 5677.5 (3) Å^3^	0.31 × 0.21 × 0.16 mm
*Z* = 4	

### Data collection

**Table:**

Bruker SMART APEXII CCD area-detector diffractometer	16557 independent reflections
Radiation source: fine-focus sealed tube	10503 reflections with *I* > 2σ(*I*)
graphite	*R*_int_ = 0.041
φ and ω scans	θ_max_ = 30.0°, θ_min_ = 2.0°
Absorption correction: multi-scan (*SADABS*; Bruker, 2005)	*h* = −18→13
*T*_min_ = 0.571, *T*_max_ = 0.741	*k* = −23→23
67215 measured reflections	*l* = −35→35

### Refinement

**Table:**

Refinement on *F*^2^	Primary atom site location: structure-invariant direct methods
Least-squares matrix: full	Secondary atom site location: difference Fourier map
*R*[*F*^2^ > 2σ(*F*^2^)] = 0.039	Hydrogen site location: inferred from neighbouring sites
*wR*(*F*^2^) = 0.121	H-atom parameters constrained
*S* = 1.02	*w* = 1/[σ^2^(*F*_o_^2^) + (0.0631*P*)^2^ + 0.2919*P*] where *P* = (*F*_o_^2^ + 2*F*_c_^2^)/3
16557 reflections	(Δ/σ)_max_ = 0.001
654 parameters	Δρ_max_ = 1.00 e Å^−^^3^
3 restraints	Δρ_min_ = −0.38 e Å^−^^3^

### Special details

**Table:**

Geometry. All e.s.d.'s (except the e.s.d. in the dihedral angle between two l.s. planes) are estimated using the full covariance matrix. The cell e.s.d.'s are taken into account individually in the estimation of e.s.d.'s in distances, angles and torsion angles; correlations between e.s.d.'s in cell parameters are only used when they are defined by crystal symmetry. An approximate (isotropic) treatment of cell e.s.d.'s is used for estimating e.s.d.'s involving l.s. planes.
Refinement. Refinement of *F*^2^ against ALL reflections. The weighted *R*-factor *wR* and goodness of fit *S* are based on *F*^2^, conventional *R*-factors *R* are based on *F*, with *F* set to zero for negative *F*^2^. The threshold expression of *F*^2^ > σ(*F*^2^) is used only for calculating *R*-factors(gt) *etc*. and is not relevant to the choice of reflections for refinement. *R*-factors based on *F*^2^ are statistically about twice as large as those based on *F*, and *R*- factors based on ALL data will be even larger.

### Fractional atomic coordinates and isotropic or equivalent isotropic displacement parameters (Å^2^)

**Table:**

	*x*	*y*	*z*	*U*_iso_*/*U*_eq_	Occ. (<1)
Ru1	0.23500 (2)	0.681950 (18)	0.163442 (13)	0.03901 (9)	
Ru2	0.04906 (2)	0.755091 (17)	0.183146 (11)	0.03391 (8)	
Ru3	0.19523 (2)	0.842925 (18)	0.130875 (12)	0.03573 (8)	
As1	0.16685 (3)	0.55179 (2)	0.182877 (16)	0.03808 (10)	
As2	−0.01950 (3)	0.64684 (2)	0.233294 (14)	0.03357 (9)	
P1	0.31142 (7)	0.91113 (6)	0.08118 (4)	0.0403 (2)	
O1	0.2778 (3)	0.6974 (2)	0.28428 (14)	0.0720 (10)	
O2	0.4536 (3)	0.6481 (3)	0.1507 (2)	0.130 (2)	
O3	0.1941 (3)	0.6619 (2)	0.04285 (14)	0.0723 (10)	
O4	−0.0261 (2)	0.6678 (2)	0.08212 (13)	0.0656 (9)	
O5	−0.1248 (3)	0.8700 (2)	0.17369 (18)	0.0905 (13)	
O6	0.1308 (3)	0.8404 (2)	0.28432 (14)	0.0721 (10)	
O7	0.3531 (2)	0.84785 (18)	0.22408 (14)	0.0652 (9)	
O8	0.0740 (2)	0.98639 (19)	0.15749 (14)	0.0685 (9)	
O9	0.0639 (3)	0.8144 (2)	0.02844 (14)	0.0824 (11)	
O10	0.6764 (3)	0.7311 (2)	0.02742 (18)	0.1004 (14)	
O11	0.4740 (3)	1.2156 (2)	0.17242 (16)	0.0889 (12)	
C1	0.2580 (3)	0.4723 (2)	0.21440 (17)	0.0481 (10)	
C2	0.2382 (4)	0.3932 (3)	0.2083 (2)	0.0716 (15)	
H2A	0.1811	0.3768	0.1884	0.086*	
C3	0.3043 (5)	0.3369 (3)	0.2320 (3)	0.0911 (19)	
H3A	0.2912	0.2833	0.2277	0.109*	
C4	0.3878 (4)	0.3614 (4)	0.2614 (3)	0.094 (2)	
H4A	0.4324	0.3245	0.2765	0.112*	
C5	0.4054 (4)	0.4390 (4)	0.2683 (3)	0.101 (2)	
H5A	0.4611	0.4555	0.2893	0.121*	
C6	0.3409 (4)	0.4949 (3)	0.2445 (2)	0.0832 (17)	
H6A	0.3546	0.5483	0.2492	0.100*	
C7	0.1002 (3)	0.4924 (2)	0.12512 (16)	0.0477 (10)	
C8	0.0060 (4)	0.4655 (3)	0.1258 (2)	0.0788 (16)	
H8A	−0.0306	0.4763	0.1554	0.095*	
C9	−0.0385 (5)	0.4225 (5)	0.0844 (3)	0.113 (3)	
H9A	−0.1046	0.4060	0.0859	0.136*	
C10	0.0131 (6)	0.4048 (4)	0.0424 (3)	0.105 (2)	
H10A	−0.0160	0.3736	0.0152	0.126*	
C11	0.1077 (7)	0.4316 (4)	0.0389 (2)	0.106 (2)	
H11A	0.1429	0.4208	0.0088	0.127*	
C12	0.1522 (5)	0.4761 (3)	0.0812 (2)	0.0844 (17)	
H12A	0.2174	0.4946	0.0792	0.101*	
C13	0.0702 (3)	0.5543 (2)	0.23829 (14)	0.0378 (8)	
H13A	0.1063	0.5544	0.2730	0.045*	
H13B	0.0300	0.5067	0.2358	0.045*	
C14	−0.1491 (3)	0.6061 (2)	0.20657 (14)	0.0384 (8)	
C15	−0.2116 (3)	0.6561 (3)	0.17702 (19)	0.0597 (12)	
H15A	−0.1909	0.7071	0.1695	0.072*	
C16	−0.3055 (4)	0.6301 (3)	0.1586 (2)	0.0799 (17)	
H16A	−0.3489	0.6643	0.1397	0.096*	
C17	−0.3347 (3)	0.5534 (3)	0.1682 (2)	0.0675 (14)	
H17A	−0.3968	0.5355	0.1546	0.081*	
C18	−0.2734 (3)	0.5047 (3)	0.19719 (19)	0.0577 (11)	
H18A	−0.2935	0.4533	0.2039	0.069*	
C19	−0.1803 (3)	0.5312 (2)	0.21701 (17)	0.0479 (10)	
H19A	−0.1388	0.4976	0.2376	0.057*	
C20	−0.0419 (3)	0.6665 (2)	0.30814 (15)	0.0383 (8)	
C21	−0.1360 (3)	0.6711 (3)	0.32606 (18)	0.0564 (12)	
H21A	−0.1911	0.6639	0.3022	0.068*	
C22	−0.1508 (4)	0.6862 (4)	0.3791 (2)	0.0784 (16)	
H22A	−0.2154	0.6891	0.3909	0.094*	
C23	−0.0700 (4)	0.6968 (4)	0.4144 (2)	0.0810 (17)	
H23A	−0.0796	0.7069	0.4501	0.097*	
C24	0.0267 (4)	0.6923 (4)	0.39655 (19)	0.0748 (16)	
H24A	0.0822	0.6998	0.4201	0.090*	
C25	0.0384 (3)	0.6768 (3)	0.34356 (17)	0.0580 (12)	
H25A	0.1028	0.6732	0.3315	0.070*	
C26	0.2623 (3)	0.9403 (3)	0.01425 (16)	0.0484 (10)	
C27	0.3035 (4)	0.9174 (3)	−0.03199 (19)	0.0682 (13)	
H27A	0.3594	0.8846	−0.0300	0.082*	
C28	0.2647 (6)	0.9414 (4)	−0.0815 (2)	0.0913 (19)	
H28A	0.2957	0.9266	−0.1122	0.110*	
C29	0.1814 (6)	0.9865 (4)	−0.0850 (2)	0.096 (2)	
H29A	0.1535	1.0009	−0.1184	0.115*	
C30	0.1369 (4)	1.0116 (4)	−0.0392 (3)	0.0881 (19)	
H30A	0.0808	1.0442	−0.0419	0.106*	
C31	0.1770 (4)	0.9876 (3)	0.0106 (2)	0.0669 (13)	
H31A	0.1469	1.0031	0.0414	0.080*	
C32	0.4268 (3)	0.8588 (3)	0.06829 (16)	0.0464 (10)	
C33	0.5216 (3)	0.8901 (3)	0.07825 (19)	0.0596 (12)	
H33A	0.5285	0.9400	0.0934	0.072*	
C34	0.6064 (3)	0.8484 (3)	0.0661 (2)	0.0695 (14)	
H34A	0.6694	0.8702	0.0737	0.083*	
C35	0.5986 (4)	0.7757 (3)	0.0429 (2)	0.0675 (14)	
C36	0.5044 (4)	0.7428 (3)	0.0333 (2)	0.0720 (15)	
H36A	0.4984	0.6928	0.0182	0.086*	
C37	0.4195 (4)	0.7835 (3)	0.0458 (2)	0.0640 (13)	
H37A	0.3569	0.7605	0.0393	0.077*	
C38	0.3562 (3)	1.0063 (2)	0.10750 (16)	0.0452 (10)	
C39	0.3977 (3)	1.0620 (3)	0.07448 (18)	0.0556 (11)	
H39A	0.3988	1.0519	0.0381	0.067*	
C40	0.4374 (4)	1.1319 (3)	0.0945 (2)	0.0633 (13)	
H40A	0.4650	1.1679	0.0716	0.076*	
C41	0.4363 (3)	1.1487 (3)	0.1486 (2)	0.0590 (12)	
C42	0.3933 (3)	1.0958 (3)	0.18175 (19)	0.0580 (12)	
H42A	0.3904	1.1071	0.2179	0.070*	
C43	0.3542 (3)	1.0254 (3)	0.16138 (17)	0.0530 (11)	
H43A	0.3257	0.9900	0.1844	0.064*	
C44	0.2585 (3)	0.6956 (2)	0.2394 (2)	0.0509 (10)	
C45	0.3716 (4)	0.6611 (3)	0.1550 (2)	0.0701 (14)	
C46	0.2049 (3)	0.6743 (3)	0.0874 (2)	0.0514 (11)	
C47	0.0074 (3)	0.7000 (3)	0.11873 (16)	0.0449 (9)	
C48	−0.0595 (3)	0.8263 (3)	0.17773 (19)	0.0528 (11)	
C49	0.1048 (3)	0.8074 (3)	0.24627 (17)	0.0478 (10)	
C50	0.2913 (3)	0.8388 (2)	0.19054 (17)	0.0446 (9)	
C51	0.1216 (3)	0.9336 (3)	0.14699 (16)	0.0457 (10)	
C52	0.1119 (3)	0.8218 (3)	0.06718 (18)	0.0513 (10)	
C53	0.7717 (5)	0.7646 (5)	0.0315 (3)	0.130 (3)	
H53A	0.8198	0.7272	0.0198	0.195*	
H53B	0.7884	0.7788	0.0680	0.195*	
H53C	0.7727	0.8110	0.0095	0.195*	
C54	0.5152 (6)	1.2734 (4)	0.1412 (3)	0.118 (3)	
H54A	0.5435	1.3144	0.1638	0.177*	
H54B	0.4639	1.2952	0.1172	0.177*	
H54C	0.5667	1.2505	0.1210	0.177*	
Cl1	0.4479 (13)	0.4905 (11)	0.0404 (6)	0.172 (6)*	0.15
Cl2	0.3550 (14)	0.5807 (10)	−0.0494 (6)	0.174 (7)*	0.15
C55	0.372 (2)	0.4939 (10)	−0.0163 (10)	0.083 (11)*	0.15
H55A	0.3065	0.4752	−0.0073	0.100*	0.15
H55B	0.3977	0.4562	−0.0411	0.100*	0.15

### Atomic displacement parameters (Å^2^)

**Table:**

	*U*^11^	*U*^22^	*U*^33^	*U*^12^	*U*^13^	*U*^23^
Ru1	0.03235 (15)	0.03420 (17)	0.05090 (19)	0.00299 (12)	0.00594 (13)	0.00324 (14)
Ru2	0.03279 (15)	0.03140 (16)	0.03801 (16)	0.00195 (12)	0.00618 (12)	0.00411 (12)
Ru3	0.03498 (16)	0.03388 (17)	0.03880 (17)	0.00010 (12)	0.00611 (12)	0.00484 (13)
As1	0.0374 (2)	0.0322 (2)	0.0447 (2)	0.00371 (16)	0.00232 (16)	−0.00020 (16)
As2	0.03317 (19)	0.0323 (2)	0.03523 (19)	0.00000 (15)	0.00221 (15)	0.00322 (15)
P1	0.0379 (5)	0.0412 (6)	0.0426 (5)	0.0010 (4)	0.0092 (4)	0.0065 (4)
O1	0.085 (3)	0.071 (2)	0.058 (2)	−0.0019 (19)	−0.0095 (19)	−0.0014 (18)
O2	0.046 (2)	0.174 (5)	0.172 (5)	0.040 (3)	0.027 (3)	0.053 (4)
O3	0.083 (3)	0.078 (3)	0.056 (2)	0.0012 (19)	0.0068 (18)	−0.0106 (18)
O4	0.063 (2)	0.083 (3)	0.0504 (19)	−0.0055 (18)	−0.0023 (16)	−0.0117 (17)
O5	0.059 (2)	0.069 (2)	0.146 (4)	0.0306 (19)	0.023 (2)	0.026 (2)
O6	0.093 (3)	0.061 (2)	0.063 (2)	−0.0188 (19)	0.0064 (19)	−0.0142 (18)
O7	0.062 (2)	0.054 (2)	0.077 (2)	−0.0101 (16)	−0.0204 (18)	0.0132 (17)
O8	0.068 (2)	0.0470 (19)	0.091 (3)	0.0143 (17)	0.0086 (18)	−0.0091 (18)
O9	0.097 (3)	0.089 (3)	0.059 (2)	−0.021 (2)	−0.020 (2)	0.0143 (19)
O10	0.083 (3)	0.093 (3)	0.130 (4)	0.045 (2)	0.050 (3)	0.020 (3)
O11	0.113 (3)	0.049 (2)	0.106 (3)	−0.028 (2)	0.014 (2)	−0.007 (2)
C1	0.045 (2)	0.040 (2)	0.059 (3)	0.0103 (18)	0.001 (2)	0.005 (2)
C2	0.061 (3)	0.042 (3)	0.110 (4)	0.004 (2)	−0.014 (3)	0.004 (3)
C3	0.081 (4)	0.041 (3)	0.149 (6)	0.009 (3)	−0.014 (4)	0.016 (3)
C4	0.072 (4)	0.068 (4)	0.139 (6)	0.023 (3)	−0.012 (4)	0.027 (4)
C5	0.079 (4)	0.066 (4)	0.150 (6)	0.009 (3)	−0.055 (4)	0.010 (4)
C6	0.081 (4)	0.043 (3)	0.120 (5)	0.006 (3)	−0.038 (3)	0.004 (3)
C7	0.063 (3)	0.034 (2)	0.046 (2)	0.0061 (19)	0.003 (2)	−0.0040 (18)
C8	0.073 (4)	0.091 (4)	0.073 (3)	−0.011 (3)	0.001 (3)	−0.037 (3)
C9	0.094 (5)	0.148 (7)	0.096 (5)	−0.026 (5)	−0.005 (4)	−0.063 (5)
C10	0.142 (7)	0.088 (5)	0.080 (5)	−0.019 (5)	−0.029 (5)	−0.023 (4)
C11	0.190 (8)	0.079 (5)	0.051 (3)	−0.001 (5)	0.020 (4)	−0.020 (3)
C12	0.115 (5)	0.074 (4)	0.066 (3)	−0.017 (3)	0.026 (3)	−0.020 (3)
C13	0.040 (2)	0.037 (2)	0.0354 (19)	0.0030 (16)	−0.0009 (16)	0.0036 (16)
C14	0.0369 (19)	0.043 (2)	0.0350 (19)	−0.0003 (16)	0.0006 (15)	0.0020 (16)
C15	0.041 (2)	0.055 (3)	0.082 (3)	−0.006 (2)	−0.008 (2)	0.022 (2)
C16	0.048 (3)	0.087 (4)	0.103 (4)	−0.003 (3)	−0.018 (3)	0.043 (3)
C17	0.040 (2)	0.079 (4)	0.081 (3)	−0.015 (2)	−0.012 (2)	0.011 (3)
C18	0.052 (3)	0.047 (3)	0.073 (3)	−0.011 (2)	−0.001 (2)	0.003 (2)
C19	0.047 (2)	0.043 (2)	0.053 (2)	−0.0032 (19)	−0.0024 (19)	0.0054 (19)
C20	0.044 (2)	0.032 (2)	0.040 (2)	−0.0016 (16)	0.0083 (17)	0.0009 (16)
C21	0.050 (3)	0.069 (3)	0.051 (3)	0.005 (2)	0.008 (2)	−0.002 (2)
C22	0.060 (3)	0.120 (5)	0.056 (3)	0.013 (3)	0.018 (3)	−0.011 (3)
C23	0.076 (4)	0.122 (5)	0.048 (3)	0.000 (3)	0.016 (3)	−0.014 (3)
C24	0.066 (3)	0.114 (5)	0.044 (3)	−0.007 (3)	−0.001 (2)	−0.012 (3)
C25	0.049 (3)	0.081 (3)	0.045 (2)	−0.002 (2)	0.009 (2)	−0.005 (2)
C26	0.045 (2)	0.051 (3)	0.049 (2)	−0.0077 (19)	0.0032 (19)	0.017 (2)
C27	0.079 (3)	0.074 (4)	0.052 (3)	0.004 (3)	0.008 (2)	0.005 (3)
C28	0.122 (5)	0.099 (5)	0.052 (3)	−0.002 (4)	−0.004 (3)	0.012 (3)
C29	0.126 (6)	0.096 (5)	0.062 (4)	−0.029 (4)	−0.026 (4)	0.027 (3)
C30	0.076 (4)	0.078 (4)	0.107 (5)	−0.002 (3)	−0.029 (4)	0.041 (4)
C31	0.063 (3)	0.064 (3)	0.073 (3)	0.005 (3)	0.000 (2)	0.023 (3)
C32	0.044 (2)	0.049 (3)	0.047 (2)	0.0102 (18)	0.0140 (18)	0.0071 (19)
C33	0.047 (2)	0.064 (3)	0.068 (3)	0.002 (2)	0.006 (2)	−0.006 (2)
C34	0.040 (2)	0.090 (4)	0.080 (4)	0.014 (3)	0.013 (2)	0.005 (3)
C35	0.068 (3)	0.066 (3)	0.071 (3)	0.021 (3)	0.032 (3)	0.020 (3)
C36	0.084 (4)	0.046 (3)	0.089 (4)	0.006 (3)	0.037 (3)	0.001 (3)
C37	0.059 (3)	0.056 (3)	0.079 (3)	−0.002 (2)	0.026 (3)	0.003 (3)
C38	0.041 (2)	0.043 (2)	0.053 (2)	0.0029 (18)	0.0133 (18)	0.0118 (19)
C39	0.060 (3)	0.054 (3)	0.054 (3)	−0.005 (2)	0.011 (2)	0.007 (2)
C40	0.068 (3)	0.049 (3)	0.075 (3)	−0.013 (2)	0.017 (3)	0.010 (2)
C41	0.058 (3)	0.045 (3)	0.075 (3)	−0.005 (2)	0.011 (2)	0.002 (2)
C42	0.066 (3)	0.052 (3)	0.057 (3)	−0.008 (2)	0.010 (2)	−0.002 (2)
C43	0.060 (3)	0.044 (3)	0.056 (3)	0.000 (2)	0.009 (2)	0.008 (2)
C44	0.049 (2)	0.038 (2)	0.065 (3)	−0.0013 (18)	−0.001 (2)	0.004 (2)
C45	0.048 (3)	0.080 (4)	0.083 (4)	0.018 (3)	0.009 (3)	0.019 (3)
C46	0.045 (2)	0.046 (3)	0.063 (3)	0.0041 (19)	0.008 (2)	0.003 (2)
C47	0.039 (2)	0.053 (3)	0.043 (2)	0.0051 (18)	0.0063 (18)	0.004 (2)
C48	0.047 (2)	0.045 (3)	0.067 (3)	0.005 (2)	0.014 (2)	0.009 (2)
C49	0.051 (2)	0.041 (2)	0.053 (3)	−0.0042 (19)	0.011 (2)	0.003 (2)
C50	0.043 (2)	0.038 (2)	0.053 (2)	−0.0007 (18)	0.0009 (19)	0.0079 (18)
C51	0.042 (2)	0.048 (3)	0.047 (2)	−0.0048 (19)	0.0031 (18)	0.0046 (19)
C52	0.058 (3)	0.049 (3)	0.047 (2)	−0.006 (2)	0.003 (2)	0.009 (2)
C53	0.075 (4)	0.167 (8)	0.154 (7)	0.058 (5)	0.042 (4)	0.031 (6)
C54	0.166 (7)	0.069 (4)	0.122 (6)	−0.048 (5)	0.029 (5)	−0.006 (4)

### Geometric parameters (Å, °)

**Table:**

Ru1—C45	1.884 (5)	C14—C15	1.376 (5)
Ru1—C44	1.929 (5)	C15—C16	1.385 (6)
Ru1—C46	1.931 (5)	C15—H15A	0.9300
Ru1—As1	2.4463 (5)	C16—C17	1.382 (7)
Ru1—Ru2	2.8469 (4)	C16—H16A	0.9300
Ru1—Ru3	2.8883 (4)	C17—C18	1.347 (6)
Ru2—C48	1.887 (4)	C17—H17A	0.9300
Ru2—C47	1.921 (4)	C18—C19	1.389 (6)
Ru2—C49	1.928 (5)	C18—H18A	0.9300
Ru2—As2	2.4335 (5)	C19—H19A	0.9300
Ru2—Ru3	2.8376 (4)	C20—C21	1.363 (5)
Ru3—C51	1.881 (5)	C20—C25	1.365 (6)
Ru3—C50	1.918 (4)	C21—C22	1.382 (6)
Ru3—C52	1.929 (5)	C21—H21A	0.9300
Ru3—P1	2.3511 (10)	C22—C23	1.368 (7)
As1—C7	1.940 (4)	C22—H22A	0.9300
As1—C13	1.952 (3)	C23—C24	1.395 (7)
As1—C1	1.953 (4)	C23—H23A	0.9300
As2—C20	1.948 (4)	C24—C25	1.374 (6)
As2—C14	1.949 (4)	C24—H24A	0.9300
As2—C13	1.974 (4)	C25—H25A	0.9300
P1—C32	1.824 (4)	C26—C27	1.369 (6)
P1—C38	1.830 (4)	C26—C31	1.392 (6)
P1—C26	1.836 (4)	C27—C28	1.380 (7)
O1—C44	1.140 (5)	C27—H27A	0.9300
O2—C45	1.130 (5)	C28—C29	1.350 (9)
O3—C46	1.140 (5)	C28—H28A	0.9300
O4—C47	1.138 (5)	C29—C30	1.390 (8)
O5—C48	1.145 (5)	C29—H29A	0.9300
O6—C49	1.143 (5)	C30—C31	1.393 (7)
O7—C50	1.155 (5)	C30—H30A	0.9300
O8—C51	1.138 (5)	C31—H31A	0.9300
O9—C52	1.140 (5)	C32—C33	1.383 (6)
O10—C35	1.361 (6)	C32—C37	1.396 (6)
O10—C53	1.394 (8)	C33—C34	1.386 (6)
O11—C41	1.364 (5)	C33—H33A	0.9300
O11—C54	1.388 (7)	C34—C35	1.363 (7)
C1—C6	1.359 (6)	C34—H34A	0.9300
C1—C2	1.373 (6)	C35—C36	1.386 (8)
C2—C3	1.410 (7)	C36—C37	1.380 (7)
C2—H2A	0.9300	C36—H36A	0.9300
C3—C4	1.366 (8)	C37—H37A	0.9300
C3—H3A	0.9300	C38—C43	1.392 (6)
C4—C5	1.346 (8)	C38—C39	1.393 (5)
C4—H4A	0.9300	C39—C40	1.381 (6)
C5—C6	1.392 (7)	C39—H39A	0.9300
C5—H5A	0.9300	C40—C41	1.388 (7)
C6—H6A	0.9300	C40—H40A	0.9300
C7—C8	1.340 (6)	C41—C42	1.370 (6)
C7—C12	1.365 (6)	C42—C43	1.388 (6)
C8—C9	1.375 (7)	C42—H42A	0.9300
C8—H8A	0.9300	C43—H43A	0.9300
C9—C10	1.329 (9)	C53—H53A	0.9600
C9—H9A	0.9300	C53—H53B	0.9600
C10—C11	1.351 (10)	C53—H53C	0.9600
C10—H10A	0.9300	C54—H54A	0.9600
C11—C12	1.406 (8)	C54—H54B	0.9600
C11—H11A	0.9300	C54—H54C	0.9600
C12—H12A	0.9300	Cl1—C55	1.6995 (10)
C13—H13A	0.9700	Cl2—C55	1.6995 (10)
C13—H13B	0.9700	C55—H55A	0.9700
C14—C19	1.367 (5)	C55—H55B	0.9700
			
C45—Ru1—C44	91.7 (2)	C16—C15—H15A	120.1
C45—Ru1—C46	91.4 (2)	C17—C16—C15	120.0 (5)
C44—Ru1—C46	175.99 (18)	C17—C16—H16A	120.0
C45—Ru1—As1	103.17 (16)	C15—C16—H16A	120.0
C44—Ru1—As1	87.29 (13)	C18—C17—C16	120.1 (4)
C46—Ru1—As1	94.46 (13)	C18—C17—H17A	120.0
C45—Ru1—Ru2	164.40 (17)	C16—C17—H17A	120.0
C44—Ru1—Ru2	82.56 (13)	C17—C18—C19	120.1 (4)
C46—Ru1—Ru2	93.79 (12)	C17—C18—H18A	120.0
As1—Ru1—Ru2	91.080 (14)	C19—C18—H18A	120.0
C45—Ru1—Ru3	108.02 (16)	C14—C19—C18	120.6 (4)
C44—Ru1—Ru3	100.56 (13)	C14—C19—H19A	119.7
C46—Ru1—Ru3	76.06 (13)	C18—C19—H19A	119.7
As1—Ru1—Ru3	147.496 (16)	C21—C20—C25	119.0 (4)
Ru2—Ru1—Ru3	59.304 (10)	C21—C20—As2	121.6 (3)
C48—Ru2—C47	93.72 (19)	C25—C20—As2	119.4 (3)
C48—Ru2—C49	91.44 (19)	C20—C21—C22	121.0 (4)
C47—Ru2—C49	174.09 (17)	C20—C21—H21A	119.5
C48—Ru2—As2	102.00 (13)	C22—C21—H21A	119.5
C47—Ru2—As2	88.23 (12)	C23—C22—C21	119.7 (5)
C49—Ru2—As2	93.51 (12)	C23—C22—H22A	120.2
C48—Ru2—Ru3	100.32 (13)	C21—C22—H22A	120.2
C47—Ru2—Ru3	92.30 (12)	C22—C23—C24	119.9 (5)
C49—Ru2—Ru3	83.93 (12)	C22—C23—H23A	120.0
As2—Ru2—Ru3	157.589 (16)	C24—C23—H23A	120.0
C48—Ru2—Ru1	160.42 (13)	C25—C24—C23	118.7 (5)
C47—Ru2—Ru1	81.75 (11)	C25—C24—H24A	120.7
C49—Ru2—Ru1	92.43 (12)	C23—C24—H24A	120.7
As2—Ru2—Ru1	96.913 (14)	C20—C25—C24	121.7 (4)
Ru3—Ru2—Ru1	61.075 (11)	C20—C25—H25A	119.1
C51—Ru3—C50	101.40 (17)	C24—C25—H25A	119.1
C51—Ru3—C52	92.56 (18)	C27—C26—C31	118.4 (4)
C50—Ru3—C52	165.87 (18)	C27—C26—P1	123.9 (4)
C51—Ru3—P1	94.76 (12)	C31—C26—P1	117.7 (3)
C50—Ru3—P1	89.91 (12)	C26—C27—C28	122.0 (5)
C52—Ru3—P1	91.12 (13)	C26—C27—H27A	119.0
C51—Ru3—Ru2	86.89 (12)	C28—C27—H27A	119.0
C50—Ru3—Ru2	93.93 (12)	C29—C28—C27	119.5 (6)
C52—Ru3—Ru2	84.57 (12)	C29—C28—H28A	120.3
P1—Ru3—Ru2	175.45 (3)	C27—C28—H28A	120.3
C51—Ru3—Ru1	143.17 (12)	C28—C29—C30	120.7 (6)
C50—Ru3—Ru1	68.93 (12)	C28—C29—H29A	119.7
C52—Ru3—Ru1	98.49 (13)	C30—C29—H29A	119.7
P1—Ru3—Ru1	119.86 (3)	C29—C30—C31	119.4 (6)
Ru2—Ru3—Ru1	59.621 (10)	C29—C30—H30A	120.3
C7—As1—C13	104.42 (17)	C31—C30—H30A	120.3
C7—As1—C1	101.18 (18)	C26—C31—C30	120.0 (5)
C13—As1—C1	98.61 (16)	C26—C31—H31A	120.0
C7—As1—Ru1	118.67 (12)	C30—C31—H31A	120.0
C13—As1—Ru1	113.06 (11)	C33—C32—C37	117.7 (4)
C1—As1—Ru1	118.13 (13)	C33—C32—P1	123.9 (4)
C20—As2—C14	102.10 (15)	C37—C32—P1	118.4 (3)
C20—As2—C13	101.59 (15)	C32—C33—C34	121.2 (5)
C14—As2—C13	105.44 (16)	C32—C33—H33A	119.4
C20—As2—Ru2	116.95 (11)	C34—C33—H33A	119.4
C14—As2—Ru2	116.29 (11)	C35—C34—C33	120.7 (5)
C13—As2—Ru2	112.70 (11)	C35—C34—H34A	119.6
C32—P1—C38	103.28 (19)	C33—C34—H34A	119.6
C32—P1—C26	103.33 (19)	O10—C35—C34	125.6 (5)
C38—P1—C26	100.68 (19)	O10—C35—C36	115.4 (5)
C32—P1—Ru3	116.51 (14)	C34—C35—C36	119.0 (4)
C38—P1—Ru3	117.01 (13)	C37—C36—C35	120.7 (5)
C26—P1—Ru3	113.89 (13)	C37—C36—H36A	119.7
C35—O10—C53	117.6 (5)	C35—C36—H36A	119.7
C41—O11—C54	119.2 (5)	C36—C37—C32	120.7 (5)
C6—C1—C2	118.8 (4)	C36—C37—H37A	119.7
C6—C1—As1	120.1 (3)	C32—C37—H37A	119.7
C2—C1—As1	121.1 (3)	C43—C38—C39	116.8 (4)
C1—C2—C3	120.1 (5)	C43—C38—P1	122.1 (3)
C1—C2—H2A	119.9	C39—C38—P1	121.1 (3)
C3—C2—H2A	119.9	C40—C39—C38	121.5 (4)
C4—C3—C2	119.7 (5)	C40—C39—H39A	119.2
C4—C3—H3A	120.2	C38—C39—H39A	119.2
C2—C3—H3A	120.2	C39—C40—C41	120.5 (4)
C5—C4—C3	119.9 (5)	C39—C40—H40A	119.8
C5—C4—H4A	120.1	C41—C40—H40A	119.8
C3—C4—H4A	120.1	O11—C41—C42	115.8 (5)
C4—C5—C6	120.6 (6)	O11—C41—C40	125.0 (4)
C4—C5—H5A	119.7	C42—C41—C40	119.2 (4)
C6—C5—H5A	119.7	C41—C42—C43	120.1 (4)
C1—C6—C5	120.9 (5)	C41—C42—H42A	120.0
C1—C6—H6A	119.6	C43—C42—H42A	120.0
C5—C6—H6A	119.6	C42—C43—C38	122.0 (4)
C8—C7—C12	117.7 (5)	C42—C43—H43A	119.0
C8—C7—As1	124.0 (3)	C38—C43—H43A	119.0
C12—C7—As1	118.3 (4)	O1—C44—Ru1	173.5 (4)
C7—C8—C9	122.5 (5)	O2—C45—Ru1	178.9 (5)
C7—C8—H8A	118.8	O3—C46—Ru1	171.8 (4)
C9—C8—H8A	118.8	O4—C47—Ru2	173.7 (4)
C10—C9—C8	119.8 (7)	O5—C48—Ru2	178.8 (4)
C10—C9—H9A	120.1	O6—C49—Ru2	174.8 (4)
C8—C9—H9A	120.1	O7—C50—Ru3	169.5 (3)
C9—C10—C11	120.4 (6)	O8—C51—Ru3	177.2 (4)
C9—C10—H10A	119.8	O9—C52—Ru3	175.4 (4)
C11—C10—H10A	119.8	O10—C53—H53A	109.5
C10—C11—C12	119.3 (6)	O10—C53—H53B	109.5
C10—C11—H11A	120.4	H53A—C53—H53B	109.5
C12—C11—H11A	120.4	O10—C53—H53C	109.5
C7—C12—C11	120.3 (6)	H53A—C53—H53C	109.5
C7—C12—H12A	119.9	H53B—C53—H53C	109.5
C11—C12—H12A	119.9	O11—C54—H54A	109.5
As1—C13—As2	113.38 (17)	O11—C54—H54B	109.5
As1—C13—H13A	108.9	H54A—C54—H54B	109.5
As2—C13—H13A	108.9	O11—C54—H54C	109.5
As1—C13—H13B	108.9	H54A—C54—H54C	109.5
As2—C13—H13B	108.9	H54B—C54—H54C	109.5
H13A—C13—H13B	107.7	Cl1—C55—Cl2	119.77 (11)
C19—C14—C15	119.5 (4)	Cl1—C55—H55A	107.4
C19—C14—As2	122.5 (3)	Cl2—C55—H55A	107.4
C15—C14—As2	117.9 (3)	Cl1—C55—H55B	107.4
C14—C15—C16	119.7 (4)	Cl2—C55—H55B	107.4
C14—C15—H15A	120.1	H55A—C55—H55B	106.9
			
C45—Ru1—Ru2—C48	57.4 (7)	C13—As1—C1—C2	82.7 (4)
C44—Ru1—Ru2—C48	126.5 (5)	Ru1—As1—C1—C2	−155.3 (4)
C46—Ru1—Ru2—C48	−51.8 (5)	C6—C1—C2—C3	−1.3 (8)
As1—Ru1—Ru2—C48	−146.4 (4)	As1—C1—C2—C3	−179.1 (4)
Ru3—Ru1—Ru2—C48	19.5 (4)	C1—C2—C3—C4	0.3 (10)
C45—Ru1—Ru2—C47	135.2 (6)	C2—C3—C4—C5	1.4 (11)
C44—Ru1—Ru2—C47	−155.73 (18)	C3—C4—C5—C6	−2.0 (11)
C46—Ru1—Ru2—C47	25.94 (18)	C2—C1—C6—C5	0.7 (9)
As1—Ru1—Ru2—C47	−68.60 (12)	As1—C1—C6—C5	178.5 (5)
Ru3—Ru1—Ru2—C47	97.27 (12)	C4—C5—C6—C1	1.0 (11)
C45—Ru1—Ru2—C49	−43.8 (6)	C13—As1—C7—C8	3.4 (5)
C44—Ru1—Ru2—C49	25.30 (17)	C1—As1—C7—C8	105.5 (4)
C46—Ru1—Ru2—C49	−153.03 (18)	Ru1—As1—C7—C8	−123.5 (4)
As1—Ru1—Ru2—C49	112.42 (12)	C13—As1—C7—C12	−176.0 (4)
Ru3—Ru1—Ru2—C49	−81.71 (12)	C1—As1—C7—C12	−73.9 (4)
C45—Ru1—Ru2—As2	−137.6 (6)	Ru1—As1—C7—C12	57.0 (4)
C44—Ru1—Ru2—As2	−68.54 (13)	C12—C7—C8—C9	0.3 (9)
C46—Ru1—Ru2—As2	113.14 (13)	As1—C7—C8—C9	−179.1 (5)
As1—Ru1—Ru2—As2	18.590 (17)	C7—C8—C9—C10	1.7 (11)
Ru3—Ru1—Ru2—As2	−175.538 (16)	C8—C9—C10—C11	−3.0 (12)
C45—Ru1—Ru2—Ru3	38.0 (6)	C9—C10—C11—C12	2.4 (12)
C44—Ru1—Ru2—Ru3	107.00 (13)	C8—C7—C12—C11	−1.0 (8)
C46—Ru1—Ru2—Ru3	−71.33 (13)	As1—C7—C12—C11	178.5 (5)
As1—Ru1—Ru2—Ru3	−165.872 (17)	C10—C11—C12—C7	−0.4 (10)
C48—Ru2—Ru3—C51	22.56 (19)	C7—As1—C13—As2	−90.9 (2)
C47—Ru2—Ru3—C51	116.77 (17)	C1—As1—C13—As2	165.1 (2)
C49—Ru2—Ru3—C51	−67.80 (18)	Ru1—As1—C13—As2	39.4 (2)
As2—Ru2—Ru3—C51	−152.28 (13)	C20—As2—C13—As1	−147.17 (19)
Ru1—Ru2—Ru3—C51	−163.97 (12)	C14—As2—C13—As1	106.6 (2)
C48—Ru2—Ru3—C50	123.78 (19)	Ru2—As2—C13—As1	−21.2 (2)
C47—Ru2—Ru3—C50	−142.01 (17)	C20—As2—C14—C19	−75.6 (3)
C49—Ru2—Ru3—C50	33.42 (18)	C13—As2—C14—C19	30.2 (4)
As2—Ru2—Ru3—C50	−51.06 (13)	Ru2—As2—C14—C19	155.8 (3)
Ru1—Ru2—Ru3—C50	−62.74 (12)	C20—As2—C14—C15	103.0 (3)
C48—Ru2—Ru3—C52	−70.3 (2)	C13—As2—C14—C15	−151.2 (3)
C47—Ru2—Ru3—C52	23.89 (18)	Ru2—As2—C14—C15	−25.5 (4)
C49—Ru2—Ru3—C52	−160.67 (19)	C19—C14—C15—C16	0.5 (7)
As2—Ru2—Ru3—C52	114.85 (14)	As2—C14—C15—C16	−178.2 (4)
Ru1—Ru2—Ru3—C52	103.16 (14)	C14—C15—C16—C17	−2.3 (8)
C48—Ru2—Ru3—Ru1	−173.47 (15)	C15—C16—C17—C18	2.4 (9)
C47—Ru2—Ru3—Ru1	−79.27 (12)	C16—C17—C18—C19	−0.7 (8)
C49—Ru2—Ru3—Ru1	96.17 (13)	C15—C14—C19—C18	1.3 (6)
As2—Ru2—Ru3—Ru1	11.69 (4)	As2—C14—C19—C18	179.9 (3)
C45—Ru1—Ru3—C51	−142.6 (3)	C17—C18—C19—C14	−1.2 (7)
C44—Ru1—Ru3—C51	−47.3 (2)	C14—As2—C20—C21	−17.1 (4)
C46—Ru1—Ru3—C51	130.5 (2)	C13—As2—C20—C21	−125.9 (4)
As1—Ru1—Ru3—C51	54.4 (2)	Ru2—As2—C20—C21	111.0 (3)
Ru2—Ru1—Ru3—C51	27.4 (2)	C14—As2—C20—C25	163.7 (3)
C45—Ru1—Ru3—C50	−61.9 (2)	C13—As2—C20—C25	54.9 (4)
C44—Ru1—Ru3—C50	33.41 (18)	Ru2—As2—C20—C25	−68.2 (4)
C46—Ru1—Ru3—C50	−148.79 (19)	C25—C20—C21—C22	0.2 (7)
As1—Ru1—Ru3—C50	135.12 (13)	As2—C20—C21—C22	−179.0 (4)
Ru2—Ru1—Ru3—C50	108.11 (13)	C20—C21—C22—C23	0.0 (9)
C45—Ru1—Ru3—C52	111.5 (2)	C21—C22—C23—C24	0.0 (10)
C44—Ru1—Ru3—C52	−153.26 (19)	C22—C23—C24—C25	−0.4 (9)
C46—Ru1—Ru3—C52	24.54 (19)	C21—C20—C25—C24	−0.6 (7)
As1—Ru1—Ru3—C52	−51.54 (14)	As2—C20—C25—C24	178.7 (4)
Ru2—Ru1—Ru3—C52	−78.55 (13)	C23—C24—C25—C20	0.7 (8)
C45—Ru1—Ru3—P1	15.24 (18)	C32—P1—C26—C27	6.0 (4)
C44—Ru1—Ru3—P1	110.52 (13)	C38—P1—C26—C27	112.6 (4)
C46—Ru1—Ru3—P1	−71.68 (13)	Ru3—P1—C26—C27	−121.3 (4)
As1—Ru1—Ru3—P1	−147.77 (4)	C32—P1—C26—C31	−175.1 (4)
Ru2—Ru1—Ru3—P1	−174.78 (3)	C38—P1—C26—C31	−68.5 (4)
C45—Ru1—Ru3—Ru2	−169.99 (18)	Ru3—P1—C26—C31	57.6 (4)
C44—Ru1—Ru3—Ru2	−74.71 (13)	C31—C26—C27—C28	1.8 (8)
C46—Ru1—Ru3—Ru2	103.10 (13)	P1—C26—C27—C28	−179.3 (4)
As1—Ru1—Ru3—Ru2	27.01 (3)	C26—C27—C28—C29	−2.5 (9)
C45—Ru1—As1—C7	−97.8 (2)	C27—C28—C29—C30	2.7 (10)
C44—Ru1—As1—C7	171.09 (19)	C28—C29—C30—C31	−2.2 (10)
C46—Ru1—As1—C7	−5.30 (19)	C27—C26—C31—C30	−1.3 (7)
Ru2—Ru1—As1—C7	88.58 (14)	P1—C26—C31—C30	179.8 (4)
Ru3—Ru1—As1—C7	65.59 (15)	C29—C30—C31—C26	1.5 (8)
C45—Ru1—As1—C13	139.4 (2)	C38—P1—C32—C33	0.5 (4)
C44—Ru1—As1—C13	48.32 (17)	C26—P1—C32—C33	105.0 (4)
C46—Ru1—As1—C13	−128.08 (17)	Ru3—P1—C32—C33	−129.3 (3)
Ru2—Ru1—As1—C13	−34.19 (12)	C38—P1—C32—C37	−177.9 (3)
Ru3—Ru1—As1—C13	−57.18 (12)	C26—P1—C32—C37	−73.3 (4)
C45—Ru1—As1—C1	25.1 (2)	Ru3—P1—C32—C37	52.4 (4)
C44—Ru1—As1—C1	−66.02 (19)	C37—C32—C33—C34	0.4 (7)
C46—Ru1—As1—C1	117.59 (19)	P1—C32—C33—C34	−177.9 (4)
Ru2—Ru1—As1—C1	−148.53 (14)	C32—C33—C34—C35	1.2 (8)
Ru3—Ru1—As1—C1	−171.52 (14)	C53—O10—C35—C34	−5.4 (8)
C48—Ru2—As2—C20	−70.3 (2)	C53—O10—C35—C36	174.0 (5)
C47—Ru2—As2—C20	−163.71 (17)	C33—C34—C35—O10	177.3 (5)
C49—Ru2—As2—C20	21.94 (18)	C33—C34—C35—C36	−2.1 (8)
Ru3—Ru2—As2—C20	104.53 (13)	O10—C35—C36—C37	−178.1 (5)
Ru1—Ru2—As2—C20	114.82 (13)	C34—C35—C36—C37	1.3 (8)
C48—Ru2—As2—C14	50.62 (19)	C35—C36—C37—C32	0.3 (8)
C47—Ru2—As2—C14	−42.82 (17)	C33—C32—C37—C36	−1.2 (7)
C49—Ru2—As2—C14	142.84 (18)	P1—C32—C37—C36	177.3 (4)
Ru3—Ru2—As2—C14	−134.58 (13)	C32—P1—C38—C43	−106.0 (4)
Ru1—Ru2—As2—C14	−124.29 (12)	C26—P1—C38—C43	147.4 (4)
C48—Ru2—As2—C13	172.52 (19)	Ru3—P1—C38—C43	23.4 (4)
C47—Ru2—As2—C13	79.09 (16)	C32—P1—C38—C39	71.5 (4)
C49—Ru2—As2—C13	−95.26 (17)	C26—P1—C38—C39	−35.1 (4)
Ru3—Ru2—As2—C13	−12.67 (13)	Ru3—P1—C38—C39	−159.1 (3)
Ru1—Ru2—As2—C13	−2.38 (12)	C43—C38—C39—C40	1.7 (7)
C51—Ru3—P1—C32	163.9 (2)	P1—C38—C39—C40	−175.9 (4)
C50—Ru3—P1—C32	62.4 (2)	C38—C39—C40—C41	−0.4 (7)
C52—Ru3—P1—C32	−103.5 (2)	C54—O11—C41—C42	−177.5 (6)
Ru1—Ru3—P1—C32	−3.02 (17)	C54—O11—C41—C40	1.5 (8)
C51—Ru3—P1—C38	41.01 (19)	C39—C40—C41—O11	179.5 (5)
C50—Ru3—P1—C38	−60.42 (19)	C39—C40—C41—C42	−1.4 (7)
C52—Ru3—P1—C38	133.7 (2)	O11—C41—C42—C43	−179.1 (4)
Ru1—Ru3—P1—C38	−125.87 (15)	C40—C41—C42—C43	1.8 (7)
C51—Ru3—P1—C26	−75.9 (2)	C41—C42—C43—C38	−0.4 (7)
C50—Ru3—P1—C26	−177.4 (2)	C39—C38—C43—C42	−1.3 (6)
C52—Ru3—P1—C26	16.7 (2)	P1—C38—C43—C42	176.3 (3)
Ru1—Ru3—P1—C26	117.18 (16)	C51—Ru3—C50—O7	−57 (2)
C7—As1—C1—C6	158.3 (4)	C52—Ru3—C50—O7	132.2 (19)
C13—As1—C1—C6	−95.0 (4)	P1—Ru3—C50—O7	38 (2)
Ru1—As1—C1—C6	27.0 (5)	Ru2—Ru3—C50—O7	−144 (2)
C7—As1—C1—C2	−23.9 (4)	Ru1—Ru3—C50—O7	160 (2)
